# A Cocoa Peptide Protects *Caenorhabditis elegans* from Oxidative Stress and β-Amyloid Peptide Toxicity

**DOI:** 10.1371/journal.pone.0063283

**Published:** 2013-05-13

**Authors:** Patricia Martorell, Esther Bataller, Silvia Llopis, Núria Gonzalez, Beatriz Álvarez, Fernando Montón, Pepa Ortiz, Daniel Ramón, Salvador Genovés

**Affiliations:** 1 Cell Biology Laboratory, Food Biotechnology Department, Biópolis SL, Paterna, Valencia, Spain; 2 Molecular Biology Laboratory, Microbial Biotechnology Department, Biópolis SL, Paterna, Valencia, Spain; Temple University, United States of America

## Abstract

**Background:**

Cocoa and cocoa-based products contain different compounds with beneficial properties for human health. Polyphenols are the most frequently studied, and display antioxidant properties. Moreover, protein content is a very interesting source of antioxidant bioactive peptides, which can be used therapeutically for the prevention of age-related diseases.

**Methodology/Principal Findings:**

A bioactive peptide, 13L (DNYDNSAGKWWVT), was obtained from a hydrolyzed cocoa by-product by chromatography. The *in vitro* inhibition of prolyl endopeptidase (PEP) was used as screening method to select the suitable fraction for peptide identification. Functional analysis of 13L peptide was achieved using the transgenic *Caenorhabditis elegans* strain CL4176 expressing the human Aβ_1–42_ peptide as a pre-clinical *in vivo* model for Alzheimer's disease. Among the peptides isolated, peptide 13L (1 µg/mL) showed the highest antioxidant activity (*P*≤0.001) in the wild-type strain (N2). Furthermore, 13L produced a significant delay in body paralysis in strain CL4176, especially in the 24–47 h period after Aβ_1–42_ peptide induction (*P*≤0.0001). This observation is in accordance with the reduction of Aβ deposits in CL4176 by western blot. Finally, transcriptomic analysis in wild-type nematodes treated with 13L revealed modulation of the proteosomal and synaptic functions as the main metabolic targets of the peptide.

**Conclusions/Significance:**

These findings suggest that the cocoa 13L peptide has antioxidant activity and may reduce Aβ deposition in a *C. elegans* model of Alzheimer's disease; and therefore has a putative therapeutic potential for prevention of age-related diseases. Further studies in murine models and humans will be essential to analyze the effectiveness of the 13L peptide in higher animals.

## Introduction

Cocoa is the unprocessed bean from the plant *Theobroma cacao*. It originated in the rain forests of the Americas and was introduced to Europe in the 16^th^ century. Historically, cocoa beans have had many uses ranging from medication to currency [1]; however, the concept that cocoa or chocolate has health benefits was not accepted throughout much of Europe until the 20^th^ century [2]. To date, numerous studies have shown that cocoa and cocoa-based products contain many different substances with beneficial effects on human health [3], [4]. Cocoa polyphenols (mainly catechin, epicatechin and procyanidins) are the major antioxidant source in the cocoa bean as they are present in high concentrations. Cocoa flavonoids are known to display antioxidant effects through the scavenging of reactive oxygen species, Fe2+ and Cu+ chelating, inhibition of key enzymes, and promoting antioxidant defenses [5]. Furthermore, there is strong evidence that consumption of polyphenol-rich cocoa products contributes to the prevention of cardiovascular diseases [3], also showing anti-tumoral properties [6] and the ability to prevent diabetes development [5]. Recently, the literature [7] has described that compounds present in cocoa powder and chocolate exert several beneficial effects on the brain, improving cognition in animals and humans. Hence, the health-promoting properties of cocoa powder have mainly been attributed to their polyphenolic compounds. However, during bean processing to produce cocoa powder, polyphenol concentration can be affected by processes like fermentation or roasting. Therefore, identification of other natural sources with antioxidative properties other than polyphenols could be an interesting aim of nutrition research.

Dietary proteins are one of the most promising bioactive compounds. Various studies have reported on the antioxidant properties of hydrolysates or bioactive peptides from plant or animal sources (for a review see [8]). These include peptides from fish processing products [9], which have been found to retard lipid peroxidation; and proteins from milk casein, bovine serum albumine, ovalbumine, soybean, oilseed proteins, wheat gliadin and maize zein, which exert antioxidative activities against the peroxidation of lipids and fatty acids [10].

The protein content of cocoa seed is 10–15% of the dry weight, with an amino acid profile rich in lysine, arginine, serine, proline, alanine, leucine, valine, phenylalanine, and methionine [11]. However, there is not much information concerning the potential beneficial properties of cocoa peptides [6] and hence to study their impact on the prevention or treatment of age-related diseases is of great interest.

Alzheimer's disease (AD) is a neurodegenerative disorder characterized pathologically by senile plaques, where the major constituent is the amyloid beta peptide (Aβ), a 39–43 amino acid peptide derived from proteolytic processing of amyloid precursor protein (APP) [12]. Alzheimer's disease is the most common type of senile dementia, whose etiology is still under discussion. Recent studies have suggested a prion-like mechanism of disease transmission through propagation of protein misfolding in animal brains [13]; however, the mechanism responsible for the initiation of Aβ accumulation is controversial. In spite of this, there is wide acceptance of the “amyloid cascade hypothesis” [14], which states that Aβ accumulation triggers downstream neurotoxic events, leading to neuronal dysfunction and death. The strongest evidence supporting this hypothesis is based on the identification of mutations in the amyloid precursor protein (APP) gene or two presenilin genes (PS1 and PS2) in familial cases of AD, which result in increased Aβ production [15]. Evidence for involvement of free radicals in AD include the presence of elevated levels of protein oxidation, lipid peroxidation products and oxidative damage to mitochondria due to the ability of Aβ peptide to act as a pro-oxidant. This knowledge indicates that the oxidation process clearly unleashes Alzheimer's disease [16].

In this context, many different *in vivo* models have been used to study the mechanism of Aβ toxicity and determine effective therapeutic strategies. More recently, different transgenic strains of the nematode *Caenorhabditis elegans* have been used as model for neurodegenerative diseases, including AD [17], [18], [19], [20]. The worm was genetically engineered to carry the human gene for Aβ42 [21], [22]. The resulting transgenic strain CL4176 develops a concomitant progressive paralysis phenotype, being a well-suited model for correlating Aβ expression and toxicity.

In the present study, a high protein cocoa by-product, namely “*Barquillo*”, obtained from the cocoa butter extraction process, was used to purify bioactive peptides with antioxidant activity and functional properties against β-amyloid peptide toxicity related to AD. The aforementioned bioactive peptides were evaluated using *in vitro* assays (inhibition of prolyl endopeptidase enzyme) and *in vivo* experiments with the model organism *C. elegans*. As a result of these assays, a promising 13-amino acid peptide was isolated, which was then used in a transcriptomic analysis in the nematode to determine the pathways it affected.

## Materials and Methods

### Sample hydrolysis

“Barquillo” is a by-product obtained from cocoa processing by pressing and rolling out cocoa butter. “Barquillo” is of high biological value due to its high protein content (20–27%). “Barquillo” was supplied by Natraceutical S.A.

“Barquillo” protein was hydrolyzed by treatment with commercial proteases: Termamyl 120L and Alcalase 2.4L FG (Novozymes, Bagsværd, Denmark).

A solution of “Barquillo” (100 g/L) was treated with the commercial proteases Alcalase and Termamyl at a final concentration of 1 µl/g each, adding CaCl_2_ (0.5 g/g). Hydrolysis was performed for one hour at 50°C in a 1 L reaction vessel equipped with stirrer (200 rpm), and stopped by boiling at 100°C for 10 min. Hydrolysates were clarified by centrifugation for 15 min at 4000 rpm, and ultrafiltrated through 0.45 µm filters to remove solid waste. Supernatant was purified to concentrate and separate the different peptides obtained after enzymatic hydrolysis.

Total protein content was determined in “Barquillo” solutions, with or without enzymatic treatment, by means of a BCA protein assay kit (Thermo Scientific, Ulm, Germany).

### Peptide purification

Peptides were concentrated by hydrophobic interaction chromatography (HIC) using the FPLC system ÄKTA Explorer (GE Healthcare, AmershaBiosciences AB) and 100 mM sodium phosphate buffer pH 7 with 1.5 M (NH_4_)_2_ SO_4_ was used as mobile phase. Hydrolyzed “Barquillo” (protease-treated) samples underwent FPLC using a Hi-Prep 16/10 Phenyl FF column (GE Healthcare, Amersham Biosciences AB). Fractions were then eluted with 100 mM sodium phosphate buffer pH 7. Peptides were purified by ultrafiltration (10 KDa Amicon Ultra, Millipore, Madrid, Spain) prior to collection. The fraction containing peptides was purified by reverse-phase chromatography (RPC). Water containing 0.1% trifluoroacetic acid (TFA)was used as mobile phase. Peptides were loaded in a Resource RPC 3 mL column (GE Healthcare, Amersham Bioscience AB, Barcelona, Spain) and eluted with a linear gradient of acetonitrile with 0.1% TFA.

Eluted peptides were identified by tandem MS after electrospray ionization (LC-ESI-MS/MS) (CBM-CSIC, Madrid, Spain).

As previously, a BCA protein assay kit was used to determine total protein content in the different fractions.

### Prolyl endopeptidase assay

Prolyl endopeptidase (PEP) activity was determined using a previously described method [23]. Reaction was performed by addition of 0.1 U/ml of PEP (Seikagaku Corporation, Tokyo, Japan) to a 100 mM phosphate buffer pH 7 containing 2 mM of Z-Gly-Pro-p-nitroaniline (Sigma, Madrid, Spain). Samples were incubated for 20 min at 30°C and then the reaction was stopped by adding a volume of 10.5% Triton X-100 in 1 M sodium acetate buffer pH 4. The amount of ρ-nitroaniline released was quantified by spectrophotometry at 410 nm. The inhibitory activity of PEP was determined by adding 100 µl of each sample obtained during the purification process to the enzymatic reaction. Z-Prolyl-Prolinal (ZPP) (0.000426 ppm, Biomol, Plymouth Meeting, USA) was used as positive control of inhibition [24].

### *C. elegans* strains and maintenance

Experiments of oxidative stress, body fat reduction and microarrays were carried out with the wild-type strain N2 (Bristol). Paralysis assays were performed with the transgenic strain CL4176 (smg-1^ts^ [pAF29(*myo-3*/Aβ_1–42_/let UTR)+pRF4(*rol-6(su10069))*]). Paralysis is induced in strain CL4176 by expression of a muscle-specific Aβ_1–42_, which depends on up-shifting temperature from 16 to 25°C [21], [22]. Mutant strain GR1321 (*tph-1*(*mg280*)) was used in body fat reduction experiments. All the strains were obtained from the *Caenorhabditis* Genetics Center (University of Minnesota).

*Caenorhabditis elegans* N2 and *tph-1* were routinely propagated on Nematode Growth Medium (NGM) plates with *Escherichia coli* strain OP50 as a food source at 20°C, while CL4176 was maintained at 16°C. Worms were synchronized by isolating eggs from gravid adults at 20°C (N2 and *tph-1*) or 16°C (CL4176), and eggs were hatched overnight in NGM plates. In the experiments, the worms were fed with the different compounds from egg through to adult stages, and transferred to new plates every two days.

### Oxidative stress assays

Worms of the N2 strain were synchronized by isolating eggs from gravid adults and hatching the eggs overnight in NG agar plates (as control media), or in NG agar plates supplemented with 100 µL of “Barquillo” solution (with or without enzymatic hydrolysis), 100 µL of peptide chromatography fractions from RPC (F8, F9 and F10) or 1 µg/mL of purified peptides (9L, 11R, 13L and 13R). Vitamin C (0.1 µg/mL) was used as an internal positive control. Assays were performed as described previously [25]. Nematodes were cultured for 7 days after egg collection. Adult nematodes (5-day adults) were harvested for acute oxidative stress treatment. Experiments were carried out in triplicate.

Viability of *C. elegans* was assessed after oxidative stress and differences between nematodes cultured in control and treatment conditions were evaluated by means of one-way analysis of variance (ANOVA) using Statgraphics plus (version 5.1) software (Manugistics, Rockville, MD).

### Paralysis assays

Strain CL4176 maintained at 16°C was egg-synchronized in the NGM plates (control medium) and NGM with the different test samples (100 µL of “Barquillo” solutions, 100 µL of peptide chromatography fractions from RPC or 1 µg/mL of purified peptides). ZPP (1 µM) was used as an internal positive control. Transgene expression was induced by up-shifting the temperature from 16°C to 25°C, starting 24 h after egg lying and maintained for 24 h. Then worms were incubated at 20°C until all the worms in the experiment became paralyzed. Paralysis was scored 24 h after induction. Paralysis in induced worms was compared with non-induced worms (maintained at 16°C until the end of the paralysis assay). Experiments were carried out in triplicate.

Statistical analysis of paralysis curves was performed using the log rank survival test provided by GraphPad Prism 4 software package.

### Measurement of Aβ_42_ aggregation in *C. elegans*

Expression of Aβ-peptide was induced in nematodes of strain CL4176, with or without treatment with 13L peptide. Worms were harvested at 49 h after temperature upshift and boiled in sample buffer (75 mM Tris-HCl pH 6.8, 3% SDS, 0.1 mg/ml bromophenol blue, 10% glycerol, 5% 2-mercaptoethanol) for 10 min, put on ice and then centrifuged 1 min at 14000 rpm. Supernatants were run on Mini Protean TGX Gels 4–15% (BioRad). Gel was transferred to Hybond-P PVDF membrane (GE Healthcare). Blot was blocked in TBS+5% blocking agent (GE Healthcare). Aβ_42_ was detected with 6E10 monoclonal antibody (Covance, Princeton, New Jersey, USA) at 1 µg/ml; secondary anti-mouse IgG peroxidase conjugate (DAKO, Glostrup, Denmark). Secondary HRP antibodies were developed in ECL Prime (GE Healthcare). Tubulin was detected with anti-tubulin antibody (Abcam, Cambridge, UK) and used as an internal control.

Dot-blot was performed by adsorbing diluted supernatants onto a nitrocellulose membrane (Bio Rad, Hercules, California, USA). Incubation with primary and secondary antibodies was performed as described for Western immunoblotting. The net intensity of each dot was analyzed with image analysis software (Molecular Imaging Software KODAK, Carestream, Rochester, New York, USA).

### Red Nile staining

The effect of 13L peptide on *C. elegans* body fat reduction was determined in wild-type strain N2 and strain GR1321 (*tph-1*(*mg280*)). Red Nile staining of lipid droplets and fluorescence quantification [26] was performed in young adult nematodes fed in control conditions (NGM) or with 1 µg/mL of 13L peptide. Experiments were carried out in triplicate. The significance of *C. elegans* body-fat reduction between control and treated conditions was analyzed by one-way analysis of variance (ANOVA) using Statgraphics plus (version 5.1) software (Manugistics, Rockville, MD).

### Gene expression analysis in *C. elegans*

Gene expression in *C. elegans* wild-type strain (N2) cultured in NGM supplemented with synthetic peptide 13L (1 µg/mL) was compared with nematodes grown in control conditions (NMG medium) at the same age (young adult worms). Synchronized populations were obtained from embryos isolated from gravid adults in the different feeding conditions. Once the worm population reached the young adult stage, samples were collected with M9 buffer, washed three times and collected in eppendorf tubes for worm disruption by sonication (3 pulses at 10 W, 20 s/pulse). Total RNA isolation was performed with RNeasy Kit (Qiagen, Hilden, Germany). RNA samples were processed for hybridization using the GeneChip® *C. elegans* Genome Array of Affymetrix (UCIM, University of Valencia). These chips contain oligonucleotide probe sets designed to asses over 22500 transcripts from the *C. elegans* genome. Four biological replicates were examined per condition by bioinformatics. Raw data obtained from Affymetrix arrays were background corrected using RMA methodology [27]. Intensity signal was standardized across arrays via quantile normalization algorithm. Gene expression analysis was conducted in order to determine mRNA differences between biological conditions. For each comparison of interest, the difference between treated samples and controls was statistically tested using limma moderated t-statistic [28]. Aiming to control the false discovery rate, p-values were corrected for multiple testing using the Benjamini and Hochberg method [29]. Finally, gene set analysis [30],[31] was carried out for each comparison using logistic regression models [32], [33].

## Results

### Hydrolysis of “Barquillo” increases the beneficial effects in the Alzheimer's disease model

Hydrolysis of “Barquillo” was performed using commercial proteases in order to increase the bioactive peptide content and study the functional effect in AD models *in vitro* and *in vivo*.

First, *in vitro* PEP inhibition was carried out in both protease-treated and non-treated “Barquillo”. Results showed a significant increase (*P≤*0.01) in the PEP inhibition percentage in the protease-treated sample (42.8±3.0 *vs.* 22.8±6.5).

**Figure 1:**
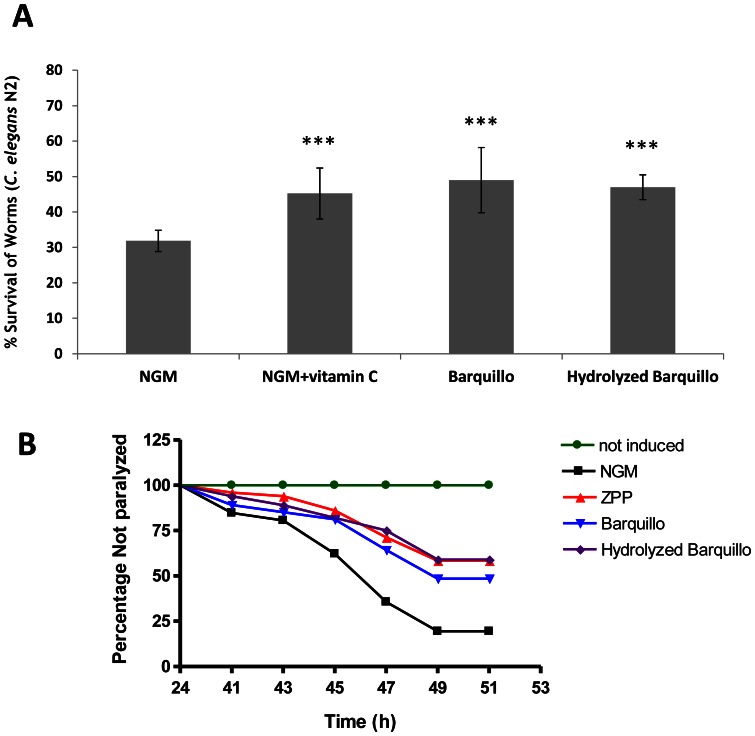
Beneficial effect of “Barquillo” increases after proteolytic treatment. (A) Percentage of survival after H_2_O_2_-induced oxidative stress in a worm population fed with “Barquillo” or protease-treated “Barquillo” samples (10 µL/mL). Vitamin C (0.1 µg/mL) was used as positive control. *** Significant at *P*≤0.001. (B) Body paralysis of *C. elegans* strain CL4176 after up-shifting temperature. ZPP (1 µM) was used as positive control. Worms without temperature-induction were included as negative control. Time refers to hours after Aβ_42_-expression induced by temperature up-shift. Worms were cultured with “Barquillo” (10 µL/mL) with or without enzymatic hydrolysis. Data are the average of three independent experiments.

After the *in vitro* analysis of “Barquillo”, we investigated its activity *in vivo* using *C. elegans* as the *in vivo* AD model. First we tested the antioxidant effect of “Barquillo” with or without proteolytic treatment on *C. elegans* wild-type strain (N2). Figure 1A shows the survival percentage after H_2_O_2_–induced oxidative stress in worm populations fed with protease-treated and non-treated “Barquillo” solutions. Results showed a significant protective effect of “Barquillo” (49% of worm survival) (*P*≤0.001) and protease-treated “Barquillo” (47% of worm survival) (*P*≤0.001) compared with control conditions (NGM) (31.8% of worm survival). Therefore, no differences in survival percentages were detected between nematodes fed with protease-treated “Barquillo” and nematodes fed with non-treated ”Barquillo”, indicating that the antioxidant effect of “Barquillo” is independent of enzymatic hydrolysis.

Furthermore, we analyzed whether protease-treated “Barquillo” rendered a higher beneficial effect than non-treated Barquillo on the *C. elegans* AD model using the transgenic strain CL4176. Figure 1B shows there was significant amelioration of body paralysis progression in “Barquillo”-fed worms compared with controls (NGM). Moreover, the observed protective effect was higher in the case of protease-treated “Barquillo”, indicating that hydrolysis with proteases could release peptides with beneficial effects against body paralysis in worms. Statistical analysis showed significant differences among paralysis curves of control and treated worms, thus indicating a delay in paralysis, especially in worms fed with protease-treated “Barquillo”. (see statistical data in Table S1)

Taking into account the beneficial effect of “Barquillo” hydrolysis in AD models, both *in vitro* and *in vivo*, subsequent purification by chromatography was performed using the protease-treated substrate.

### Purified chromatography fractions obtained from proteolysis of “Barquillo” have beneficial effects on Alzheimer's disease model

After hydrolysis of “Barquillo”, 150 mL of sample supernatant was taken for further purification. This volume was diluted in water and loaded onto a Hi-Prep 16/10 Phenyl FF hydrophobic interaction column. Eluted peaks with 100 mM sodium phosphate buffer pH 7 were monitored at 280 nm, and each fraction was collected in 10 mL. The collected chromatographic fractions were tested for the *in vitro* PEP inhibition assays.

PEP inhibition and total protein were measured in ultrafiltrated (10 KDa) fractions (Table 1). Results indicated that fraction F4 displayed the highest PEP inhibition activity; therefore, it was selected for further purification by RPC. The total volume of F4 (HIC) was loaded into a Resource RPC 3 ml column and finally peptides were eluted with a linear gradient 0.1% acetonitrile TFA. All RPC fractions obtained were *in vitro* assayed, and those samples with the higher inhibitory effect upon PEP activity were selected. Table 1 shows the RPC fractions with the highest inhibitory activity. These fractions were used to study their effect on the AD *C. elegans* model.

**Table 1:** Peptide purification steps by HIC and RPC from Hydrolyzed “Barquillo” (protease-treated).

Purification step	PEP inhibitory activity (%)	Total protein (g)	Yield (%)
Hydrolyzed “Barquillo”	42.8±3.0	0,837	100
F4 (HIC)	35.4±17.4	0,070	8.36
F8 (RPC)-F4 (HIC)	34.5±2.3	0.032	3.82
F9 (RPC)-F4 (HIC)	39.0±1.7	0.018	2.15
F10 (RPC)-F4 (HIC)	26.5±3.4	0.009	1.08

**Figure 2:**
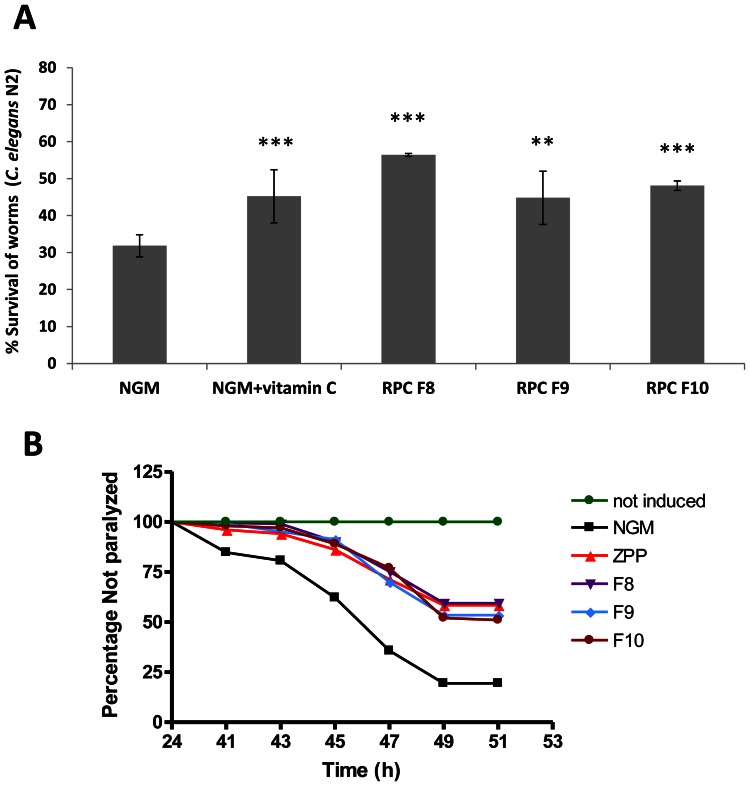
Reverse chromatography fractions F8, F9 and F10 obtained during a purification process have antioxidant properties and beneficial effects in the *C.elegans* Alzheimer's model. (A) Antioxidant effect of RPC fractions, in terms of percentage of live worms after oxidative stress. Vitamin C (0.1 µg/mL) was used as positive control. *** Significant at *P*≤0.001; ** Significant at *P*≤0.01. (B) Effect of RPC fractions on body paralysis delay in *C. elegans* CL4176. ZPP (1 µM) was used as positive control. Worms without temperature-induction were included as negative control. Data are the average of three independent experiments.

First, the protective effect upon oxidative stress in the strain N2 of *C. elegans* was assayed with the RPC fractions F8, F9 and F10 obtained from HIC F4 fraction. Figure 2A shows that all reverse phase chromatography fractions are able to increase the worm's survival after induction of oxidative stress with hydrogen peroxide (2 mM). Among them, fraction F8 showed the highest antioxidant effect, with 56.4% of worm survival (*P*≤0.001). This purified extract provided a higher protective effect than the original hydrolyzed “Barquillo” (47%), indicating that proteolysis and purification processes increased the beneficial effect of the by-product.

We also examined whether the selected RPC fractions (F8, F9 and F10) reduced nematode body paralysis using transgenic strain CL4176. Addition of these fractions to the agar media (10 µL/mL) significantly reduced the percentage of paralyzed worms at each measuring point (Figure 2B). Specifically, fraction F8 delayed the onset of paralysis in worms induced to express Aβ_42_ (Table S1). We found that the majority of nematodes cultured in control conditions (NGM) were paralyzed (80.3%) after 49 h of Aβ_42_ peptide induction, while only 40%, 46.8% or 47.62% of nematodes feeding on fraction F8, F9 or F10 were paralyzed by this time.

Taking into account the antioxidant activity and protective effect of RPC fractions F8, F9 and F10 on *in vivo* paralysis, further studies were focused on identifying their peptide profiles by LC-ESI-MS/MS.

### Purified peptides have *in vivo* antioxidant effect and potential benefits for AD

**Figure 3:**
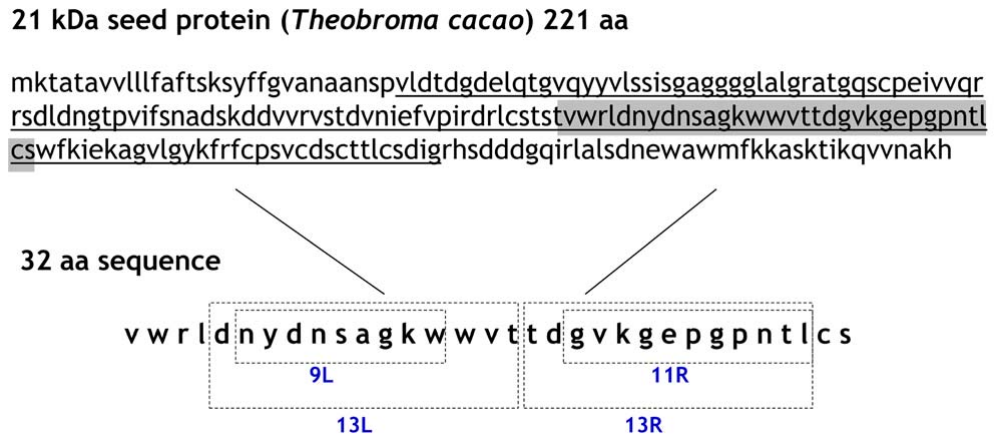
Identification of four selected peptides included in the 21 kDa seed protein from *Theobroma cacao*. Trypsin inhibitor protein sequence is underlined.

RPC fractions F8, F9 and F10 from HIC F4 fraction were selected in order to identify potential functional peptides. A total of 3, 7 and 7 peptides were identified in fractions F8, F9 and F10, respectively (Table 2). All these peptides belonged to the 21 kDa seed protein from *Theobroma cacao*. We determined that most of these peptides had identical sequence homology with the region encoding trypsin inhibitor activity, specifically in a short sequence of 32 amino acids (Figure 3). As this region has a protease inhibitor function, we focused on this sequence for peptide selection to further investigate whether the selected peptides also showed PEP inhibition and beneficial properties in the *in vivo* AD model.

**Table 2:** Peptide identification by LC-ESI-MS/MS of RPC fractions from F4 HIC.

	Molecular Weight (Da)	Sequence	Origin Protein
**F8 RPC**	1067,37	D.GVKGEPGPNTL.C	21 kDa seed protein
	1283,41	T.TDGVKGEPGPNTL.C	21 kDa seed protein
	1483,69	W.VTTDGVKGEPGPNTL.C	21 kDa seed protein
**F9 RPC**	1017,33	H.SDDDGQIRL.A	21 kDa seed protein
	1053,31	D.NYDNSAGKW.W	21 kDa seed protein
	1168,41	L.DNYDNSAGKW.W	21 kDa seed protein
	1283,53	T.TDGVKGEPGPNTL.C	21 kDa seed protein
	1437,47	W.RLDNYDNSAGKW.W	21 kDa seed protein
	1483,67	W.VTTDGVKGEPGPNTL.C	21 kDa seed protein
	1857,83	A.ANSPVLDTDGDELQTGVQ.Y	21 kDa seed protein
**F10 RPC**	1053,35	D.NYDNSAGKW.W	21 kDa seed protein
	1168,35	L.DNYDNSAGKW.W	21 kDa seed protein
	1283,57	T.TDGVKGEPGPNTL.C	21 kDa seed protein
	1554,67	L.DNYDNSAGKWWVT.T	21 kDa seed protein
	1669,41	W.WVTTDGVKGEPGPNTL.C	21 kDa seed protein
	1857,85	A.ANSPVLDTDGDELQTGVQ.Y	21 kDa seed protein
	2020,93	A.ANSPVLDTDGDELQTGVQY.Y	21 kDa seed protein

A total of four peptides were selected from this 32 amino-acid sequence (Figure 3). Among these peptides, 9L (NYDNSAGKW) and 11R (GVKGEPGPNTL) were selected as the minimum common sequence on both sides of this sequence, left and right. In addition, longer peptides containing these 9L or 11R sequences were also chosen, 13L (DNYDNSAGKWWVT) and 13R (TDGVKGEPGPNTL), respectively. An *in vitro* study of these peptides showed their inhibitory activity (IC_50_) against PEP enzyme. Lower values, 0.51; 0.19 mg/mL were displayed by 9L and 13L peptides, respectively, while peptides 11R and 13R showed >1.48 mg/mL as IC_50_ value.

**Figure 4:**
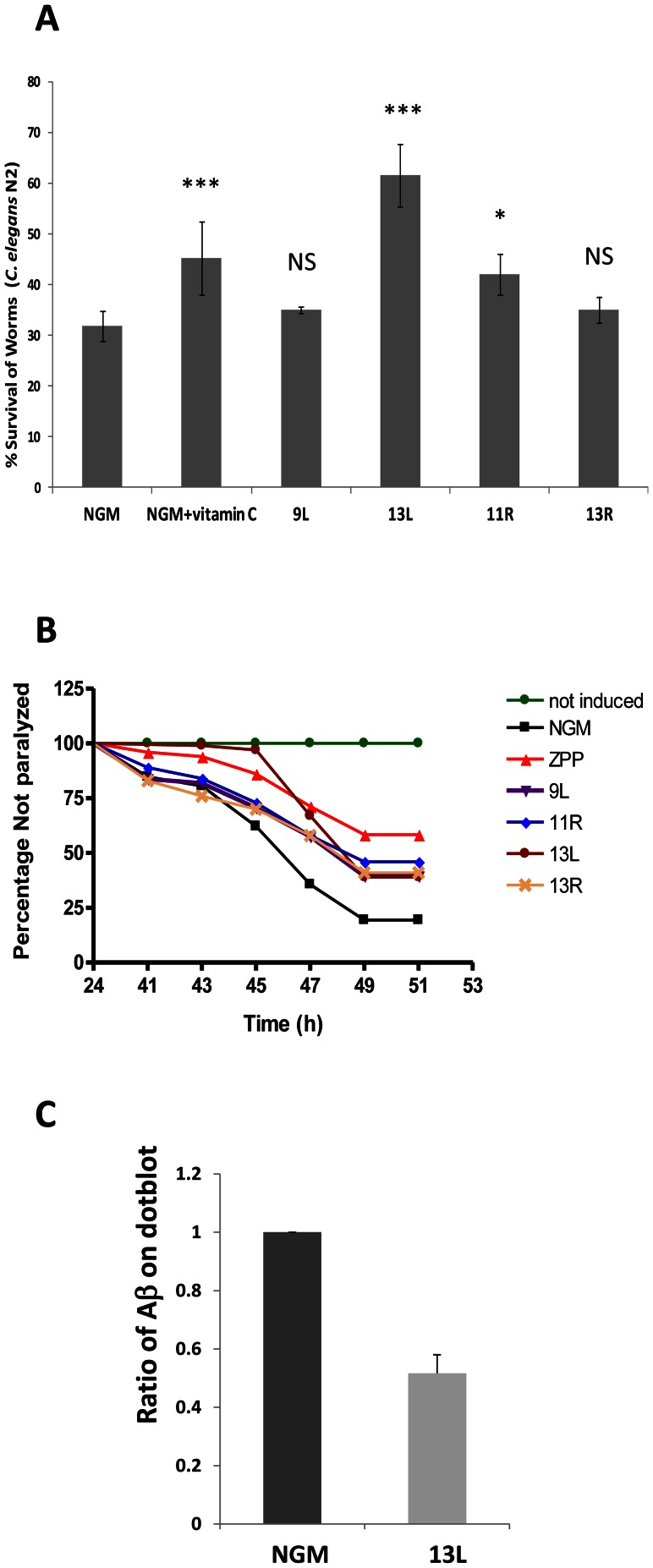
*In vivo* properties of synthetic peptides in *C.elegans*. (A) Antioxidant activity tested in *C. elegans* wild-type strain (N2) was noteworthy for 13L peptide. *** Significant at *P*≤0.001; * Significant at *P*≤0.05; NS: No significant differences with respect to the control (NGM). (B) Body paralysis of *C. elegans* CL4176 measured after temperature up-shift in nematodes fed with 1 µg/mL of the different purified peptides (9L, 11R, 13L and 13R). Data are the average of three independent experiments. (C) Effect of 13L peptide on Aβ accumulation in *C. elegans* CL4176. Net intensity quantification of dot blot analysis of total protein preparations (20 ng/dot) from *C. elegans* CL4176 on NGM or NGM+13L peptide; net intensity of non induced control was subtracted. Error bars = SEM.

These synthesized peptides (9L, 11R, 13L and 13R) were analyzed to study their antioxidant effect in *C. elegans*. Figure 4A shows that two peptides, 9L and 13R, did not produce a significant effect on worm survival rates. Conversely, peptides 13L and 11R produced significant protection upon oxidative stress (*P*≤0.001 and *P*≤0.05, respectively), especially in the case of nematodes fed on 13L, with 64.35% survival. These results indicate a marked antioxidant effect of the purified 13L peptide, obtained from RPC fraction F10, which was higher than the initial pre-purified samples of “Barquillo”.

We further tested whether the selected purified peptides also produced a significant reduction in CL4176 body paralysis. Figure 4B shows results for body paralysis in nematodes fed with these peptides. All studied peptides delayed body paralysis onset compared with nematodes cultured in control conditions (NGM). The effects of 9L, 11R and 13R were very similar to each other (*P* = 0.0029; *P* = 0.0002 and *P* = 0.0028 respectively) (Table S1). However, peptide 13L delayed paralysis onset and produced a markedly higher effect between 24 h and 47 h after Aβ_42_ induction (*P*≤0.0001), concluding that the most protective effect was provided by 13L peptide.

### The 13L peptide reduces Aβ aggregate in the *C. elegans* transgenic strain CL4176

To determine whether 13L peptide delayed body paralysis onset in CL4176 by reducing expression of the Aβ transgene, Aβ deposits were monitored by western immunoblotting after 49 hours of induction (Figure S1). 13L peptide treatment resulted in a reduction in total Aβ levels analysed by dot blot using specific Aβ antibodies. Quantification of the dots by image analysis shown a 50% of reduction of Aβ deposits after treatment with 13L (Figure 4C)

### The 13L peptide promotes up-regulation of pyruvate metabolism and proteasomal functions in *C. elegans*

We analyzed the effect of 13L peptide on *C. elegans* (N2) gene expression using microarray experiments. Four biological replicates of *C. elegans* fed with the synthetic peptide 13L (1 µg/mL) were compared with nematodes cultured in control conditions. RNA was isolated from nematodes and used for hybridization to Affymetrix *C. elegans* arrays. Microarray data are available through the NCBI Gene Expression Omnibus (GEO) data repository under accession GSE44318 (http://www.ncbi.nlm.nih.gov/geo/).

Firstly, differentially expressed genes after 13L treatment were studied in nematodes fed with this peptide. The first analysis focused on a set of genes selected according to their fold-change value. We selected the 10 most up-regulated genes in the nematodes fed with 13L peptide compared with nematodes cultured under control fed conditions. Fold-change values of these genes are described in Table S2. We determined the induction of two genes. The first was *FSHR*-1, the mammalian follicle stimulatory hormone receptor homolog, which is a putative neuropeptide receptor required for cholinergic neurotransmission. The second gene was *SNF*-5, which belongs to the sodium-neurotransmitter symporter family. These results could suggest that 13L peptide activity could be related with synaptic function in the nematode.

Furthermore, two different databases, GO Biological Process and KEGG Pathways (Kioto encyclopedia of genes and Genomes), were used to establish functional classes that were up-regulated in the nematodes treated with 13L peptide. Only two biological processes were found to be significantly up-regulated (*P≤*0.05) in treated worms, and these were related with cell division, specifically cytokinesis (GO:0000910), and organic-acid metabolism (GO: 0019752).

Further data analysis focused on the metabolic pathways affected by the 13L peptide. Thirteen different KEGG pathways were identified to be significantly up-regulated (*P≤*0.05) under treated conditions (Table 3). Pyruvate metabolism was the most significantly up-regulated metabolic pathway determined in nematodes fed with 13L peptide. The list also included other amino acid metabolism/degradation pathways, carbohydrate and fatty acid oxidation (citrate cycle), development and protein degradation (proteasome).

**Table 3:** Up-regulated KEGG pathways in nematodes treated with 1 µg/mL of 13L peptide.

Significant up-regulated KEGG Pathways		p-value
Gene ID	Description	
00620	Pyruvate metabolism	0.002
00640	Propanoate metabolism	0.004
04320	Dorso-ventral axis formation	0.004
00280	Valine, leucine and isoleucine degradation	0.012
00650	Butanoate metabolism	0.020
00380	Tryptophan metabolism	0.022
00450	Seleno amino acid metabolism	0.022
03050	Proteasome	0.035
00900	Terpenoid backbone biosynthesis	0.040
00250	Alanine, aspartate and glutamate metabolism	0.040
00910	Nitrogen metabolism	0.040
00020	Citrate cycle (TCA cycle)	0.049
00270	Cysteine and methionine metabolism	0.049

## Discussion

The present study has shown how a cocoa by-product, “Barquillo”, exhibits antioxidant properties and the ability to reduce paralysis resulting from Aβ_1–42_ peptide expression in a transgenic *C. elegans* strain. Interestingly, protein hydrolysis of “Barquillo” enhanced the antioxidative effect and protective activity against Aβ toxicity in the nematode. This could be explained by the release of bioactive peptides from the protein fraction of the by-product. These results support the important role of other compounds (other than polyphenols) in the antioxidant activity of cocoa and chocolate [5]. Therefore, a purification process was performed in order to isolate and identify these bioactive peptides. Fractionation of hydrolyzed “Barquillo” resulted in three RPC fractions with antioxidant properties and the ability to delay paralysis in the *C. elegans* AD model. All the identified peptides in these fractions belonged to the 21 kDa seed protein from *Theobroma cacao* and, surprisingly, many of them were located in a region encoding trypsin inhibitor activity. The selected peptides, 9L (NYDNSAGKW), 11R (GVKGEPGPNTL), 13L (DNYDNSAGKWWVT) and 13R (TDGVKGEPGPNTL), also displayed *in vivo* antioxidant activity and the ability to ameliorate Aβ-induced paralysis in *C. elegans*. Specifically the 13L peptide provided most protection against oxidative stress, producing a significant delay in nematode paralysis. Additionally, an important reduction in the Aβ peptide deposit was observed in 13L peptide-fed nematodes. These results would suggest that the protective effect of 13L peptide is mediated by inhibiting Aβ oligomerization, which is directly associated with Aβ-toxicity in the *C. elegans* transgenic model [34]. A similar effect has previously been reported for *Ginkgo biloba* extract EGb 761 [17], [34] and glycitein from soybeans [35]. Other research into a coffee extract and an antihypertensive drug (reserpine) has also showed an effect on delaying paralysis; however, no effects were observed in Aβ peptide expression levels [36], [37].

To understand the molecular mechanisms underlying the 13L peptide protection in *C. elegans*, differential gene expression was analyzed by DNA arrays. Pyruvate metabolism was the most significant up-regulated metabolic pathway in peptide 13L-fed nematodes. This pathway is considered the output of glycolysis and the metabolic intersection of different pathways (such as propanoate metabolism, butanoate metabolism, leucine and lysine biosynthesis and citrate cycle). In this metabolic pathway, pyruvate is decarboxylated by pyruvate dehydrogenase complex (PDH) to produce acetyl-coA, which is the key substance for ATP synthesis (through citrate cycle) and synthesis of acetylcholine. Furthermore, previous reports have shown that PDH inactivation results in acetylcholine reduction in cholinergic neurons, and therefore, neuronal death as a consequence of energy production failure [38]. Also, PDH reduction is related with glycolysis reduction and mitochondrial dysfunction, which are described to be present in AD [39], [40], [41], [42]. The microarray results indicated an up-regulation of pyruvate metabolism (and other related-pathways such as propanoate and butanoate metabolism), which could lead to an increase in acetyl-CoA production. This finding suggests that up-regulation of pyruvate metabolism as the central pathway could also up-regulate related metabolic pathways, leading to increased carbohydrate metabolism, including glucose. Furthermore, we determined citrate-cycle up-regulation after nematodes ingested 13L peptide. In the human brain, reduced activity of some tricarboxylic acid cycle (TCA) enzymes has been described in AD patients [39]. Taking into account that the major portion of glucose flux takes place through the TCA cycle [41], our results would indicate that 13L peptide could enhance the glucose metabolism to ensure ATP demands for neuronal cells.

**Figure 5:**
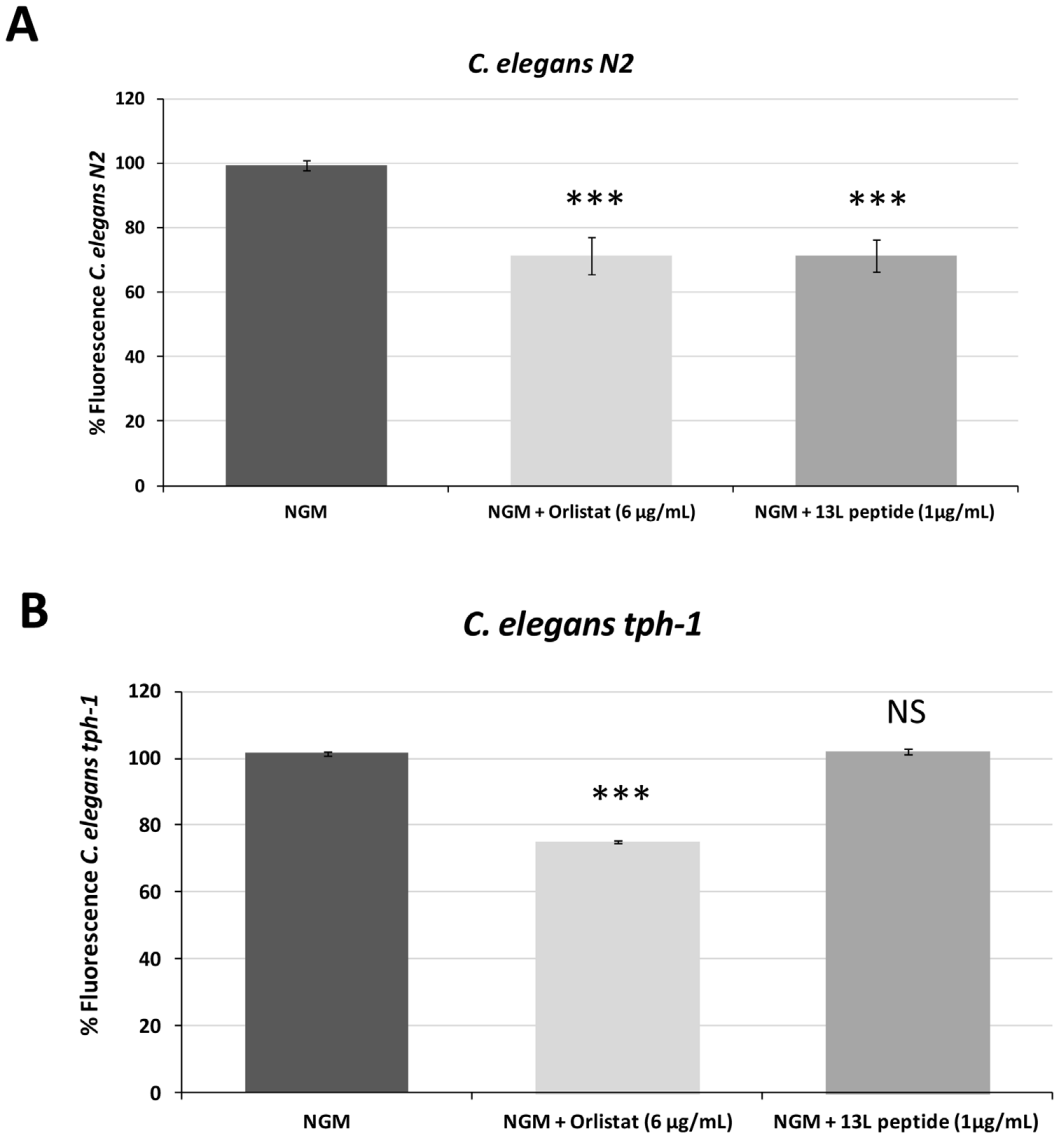
Tryptophan metabolism pathway is a molecular target of 13L peptide. (A) Body fat reduction in *C. elegans* wild-type strain N2 provided by 13L peptide feeding. (B) 13L peptide did not produce fat reduction in the mutant strain *tph-1*. *** Significant at *P*≤0.001; NS: No significant differences with respect to the control (NGM).

Worm culture supplementation with 13L peptide also up-regulated tryptophan metabolism in the nematodes. Tryptophan is an essential amino acid for humans with two metabolic pathways: the methoxyindole pathway, which leads to formation of serotonin (5-HT) and melatonin, and the kynurenine pathway, which is involved in the cleavage of the indol ring of tryptophan to produce kynurenine [43]. In *C. elegans*, synthesized serotonin from tryptophan has important functions in both the nervous system and other tissues, and is also involved in behaviors such as egg laying, pharyngeal pumping, male mating, and experience-dependent regulation of locomotion [44]. From microarray data, we found that several genes within the tryptophan metabolism pathway were up-regulated (with positive fold change). This was the case of the tyrosine decarboxylase (*tdc-1*), which is required for octopamine synthesis from tyramine, a putative neurotransmitter necessary to coordinate locomotion in *C. elegans* [45]. In addition, the L-amino acid/L-histidine decarboxylase (C05D2.3) was positively regulated within the tryptophan metabolic pathway, probably leading to serotonin synthesis. Finally, other hypothetical proteins showed positive regulation within this pathway, and were enzymes involved in the synthesis of acetyl-coA, substrate of glycolysis (2-oxoglutarate dehydrogenase (T22B11.5); acetyl-coA acetyltransferase (T02G5.4); hydroxyacyl-coA dehydrogenase, (T08B2.7). These proteins are also involved in the regulation of adiposity in *C. elegans* [46]. Furthermore, the tryptophan metabolism pathway was confirmed to be a molecular target of 13L peptide by means of experiments with the *C. elegans* mutant strain for the tryptophan hydroxylase TPH-1, which is essential for serotonin biosynthesis. Animals bearing a TPH-1 deletion mutation do not synthesize serotonin and *tph-1* mutants display abnormalities in behavior and metabolism, such as large amounts of stored fat [47]. Our study showed that 13L peptide produced significant body fat reduction in the wild-type strain of *C. elegans* (N2) (Figure 5A). However, this phenotype was not observed in the *tph-1* mutant strain (Figure 5B), suggesting that tryptophan metabolism is a mechanism of 13L peptide function.

One of the most interesting up-regulated pathways in 13L peptide-treated nematodes was the proteasome. Proteasome is a large protein complex whose function is to degrade ubiquitinated proteins by an ATP-dependent mechanism [48]. Genetic studies have previously indicated that protein homeostasis is a major factor affecting longevity [49]. The interruption of this homeostasis can result in protein misfolding and in the accumulation of insoluble protein fibrils and aggregates, such as amyloids. Thus, the search for compounds or molecules that maintain protein homeostasis could have a beneficial effect against ageing and age-related diseases. Our results clearly indicate the effect of 13L peptide on proteosomal functions; therefore, it could be contributing to protein homeostasis in the nematode. In addition, some studies have provided evidence that proteosome could contribute directly to AD by modulating presenilins and, therefore, maturation of β-amyloid precursor [50]. Other authors have demonstrated the proteosome regulatory function of AIP-1 and its effect on Aβ-accumulation and Aβ-paralysis reduction [51]. We found various genes within the up-regulated proteosome pathway encoding proteasome regulatory particles after 13L peptide ingestion. One group comprised *RPN* genes, which encode a non-ATPase subunit of the 26S proteasome 19S regulatory particles (RP) base subcomplex and are involved in unfolding and recognition of protein substrates and/or recycling ubiquitin moieties during protein degradation. The other regulatory genes were *RPT* genes, which are believed to be involved in unfolding protein substrates and translocating them into the core proteolytic particle (CP) of the proteasome. Therefore, this could indicate that 13L peptide-fed nematodes have a cellular response to regulate proteosomal functions, enhancing protein degradation and protein turnover.

**Figure 6:**
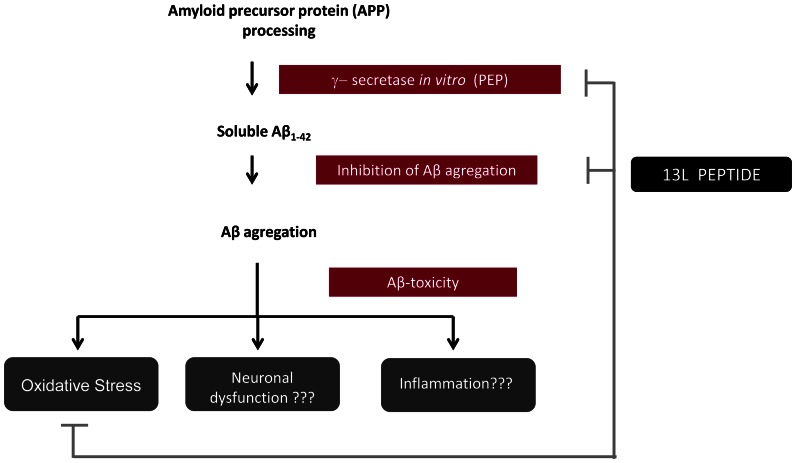
Proposed model for 13L peptide mechanism. Our working hypothesis is that 13L peptide could act through repression of the main events present in AD: oxidative stress, neuronal dysfunction and inflammation. Our study shows that 13L peptide mechanisms reducing Aβ toxicity are a combined effect of PEP inhibition activity, antioxidant properties and ability to inhibit Aβ-aggregation.

In summary, a new bioactive peptide (13L) with antioxidant activity and beneficial properties against AD has been described in this study. This peptide was obtained from a cocoa by-product, but has also been identified in different commercial chocolate samples (data not shown). Our results suggest a mechanism based on the activity of 13L peptide at different levels (Figure 6). Firstly, the ability of 13L peptide to inhibit PEP activity *in vitro*, which is described as a putative γ-secretase [52] and which has a *C. elegans* homolog [53]; secondly, the *in vivo* effect of 13L peptide on Aβ-aggregation inhibition and thirdly, Aβ-toxicity reduction through antioxidant activity and modulation of synaptic and proteosomal functions.

Therefore, this study provides evidence for the potential therapeutic use of 13L peptide in the prevention of aging-related diseases. Further studies with murine models and human trials will be essential in order to validate the effectiveness of this peptide.

## Supporting Information

Figure S1 Effect of 13L peptide on Aβ accumulation in *C. elegans* CL4176. Immunoblot assay of Aβ deposits 49 h after up-shift of CL4176 on NGM or NGM + 13L peptide plates. Total protein preparations (20 mg/lane) were run on a 4–15% polyacrylamide SDS gel and probed with anti-Aβ monoclonal antibody 6E10. Tubulin immunoreactivity is shown as loading control.
(TIF)

Table S1 Statistical analysis of paralysis curves obtained in CL4176 worms fed with “Barquillo” samples (with or without protein hydrolysis), purified chromatography fractions (RPC) and purified peptides. Analysis was performed with paired log rank survival test using Graphpad Prism v.4 software. Mid paralysis indicate the time (h) when the 50% of worms were paralyzed.
(DOC)

Table S2 Fold-change values of the 10 most up-regulated genes in nematodes fed with 13L peptide.
(DOC)
