# Supplementary material for: A Cocoa Peptide Protects Caenorhabditis elegans from Oxidative Stress and β-Amyloid Peptide Toxicity
Source: PLoS One. 2013 May 13;8(5):e63283. doi: 10.1371/journal.pone.0063283 (PMC3652819; doi:10.1371/journal.pone.0063283)
Supplement: Table S1 — Statistical analysis of paralysis curves obtained in CL4176 worms fed with “Barquillo” samples (with or without protein hydrolysis), purified chromatography fractions (RPC) and purified peptides. Analysis was performed with paired log rank survival test using Graphpad Prism v.4 software. Mid paralysis indicate the time (h) when the 50% of worms were paralyzed. (DOC) [file pone.0063283.s002.doc]

| **Treatment** | **Onset Paralysis*** | **Mid Paralysis (h)** | **Log Rank *X*^2^** | ***P*-value** |
| --- | --- | --- | --- | --- |
| NGM | 41 h/85.8% | 47 h |  |  |
| NGM + ZPP | 41 h/96.7% | >49h | 35.46 | *P*≤ 0.0001 |
| NGM + “Barquillo” | 41 h/ 89.7% | 49 h | 20.03 | *P*≤ 0.0001 |
| NGM + Hydrolyzed “Barquillo” | 41 h/ 94.0% | >49h | 37.81 | *P*≤ 0.0001 |
| NGM + F8 (RPC)-F4(HIC) | 43 h/ 99.5% | >49h | 42.27 | *P*≤ 0.0001 |
| NGM + F9 (RPC)-F4(HIC) | 41 h/ 99.5% | >49h | 34.68 | *P*≤ 0.0001 |
| NGM + F10 (RPC)-F4(HIC) | 41 h/ 98.7% | >49h | 33.78 | *P*≤ 0.0001 |
| NGM + 9L peptide | 41 h/ 84.8% | 49 h | 8.857 | *P=* 0.0029 |
| NGM + 11R peptide | 41 h/ 89.9% | 49 h | 13.43 | *P=* 0.0002 |
| NGM + 13L peptide | 43 h/ 99.1% | 49 h | 24.01 | *P*≤ 0.0001 |
| NGM + 13R peptide | 41 h/83.4% | 49 h | 8.93 | *P=* 0.0028 |

**Supplementary Table S1**

*****Time (h) of onset paralysis and % of worms non paralyzed is showed.
