# Supplementary material for: A Cocoa Peptide Protects Caenorhabditis elegans from Oxidative Stress and β-Amyloid Peptide Toxicity
Source: PLoS One. 2013 May 13;8(5):e63283. doi: 10.1371/journal.pone.0063283 (PMC3652819; doi:10.1371/journal.pone.0063283)
Supplement: Table S2 — Fold-change values of the 10 most up-regulated genes in nematodes fed with 13L peptide. (DOC) [file pone.0063283.s003.doc]

| **Symbol** | **Gene Name** | **Fold Change** |
| --- | --- | --- |
| sel-7 | Suppressor/Enhancer of Lin-12 | 0.39 |
| msi-1 | MuSashI (fly neural) family | 0.40 |
| F35F10.12 | Hypothetical protein | 0.41 |
| Y37D8A.17 | Hypothetical protein | 0.40 |
| C33E10.5 | Hypothetical protein | 0.40 |
| fshr-1 | FSHR | 0.49 |
| R148.3 | Hypothetical protein | 0.45 |
| snf-5 | Sodium:Neurotransmitter symporter Family | 0.39 |
| T21D12.7 | Hypothetical protein | 0.41 |
| rgs-6 | Regulator of G protein Signaling | 0.44 |

**Supplementary Table S2**

* Mammalian follicle stimulating hormone receptor homolog
